# Assessment of Ablative Margin After Microwave Ablation for Hepatocellular Carcinoma Using Deep Learning-Based Deformable Image Registration

**DOI:** 10.3389/fonc.2020.573316

**Published:** 2020-09-24

**Authors:** Chao An, Yiquan Jiang, Zhimei Huang, Yangkui Gu, Tianqi Zhang, Ling Ma, Jinhua Huang

**Affiliations:** ^1^Department of Minimal Invasive Intervention, State Key Laboratory of Oncology in South China, Collaborative Innovation Center for Cancer Medicine, Sun Yat-sen University Cancer Center, Guangzhou, China; ^2^College of Software, Nankai University, Tianjin, China

**Keywords:** microwave ablation, deep learning-based deformable image registration, ablative margin, hepatocellular carcinoma, local tumor progression

## Abstract

**Aim:** To assess the ablative margin (AM) after microwave ablation (MWA) for hepatocellular carcinoma (HCC) with a deep learning-based deformable image registration (DIR) technique and analyze the relation between the AM and local tumor progression (LTP).

**Patients and Methods:** From November 2012 to April 2019, 141 consecutive patients with single HCC (diameter ≤ 5 cm) who underwent MWA were reviewed. Baseline characteristics were collected to identify the risk factors for the determination of LTP after MWA. Contrast-enhanced magnetic resonance imaging scans were performed within 1 month before and 3 months after treatment. Complete ablation was confirmed for all lesions. The AM was measured based on the margin size between the tumor region and the deformed ablative region. To correct the misalignment, DIR between images before and after ablation was achieved by an unsupervised landmark-constrained convolutional neural network. The patients were classified into two groups according to their AMs: group A (AM ≤ 5 mm) and group B (AM > 5 mm). The cumulative LTP rates were compared between the two groups using Kaplan–Meier curves and the log-rank test. Multivariate analyses were performed on clinicopathological variables to identify factors affecting LTP.

**Results:** After a median follow-up period of 28.9 months, LTP was found in 19 patients. The mean tumor and ablation zone sizes were 2.3 ± 0.9 cm and 3.8 ± 1.2 cm, respectively. The mean minimum ablation margin was 3.4 ± 0.7 mm (range, 0–16 mm). The DIR technique had higher AUC for 2-year LTP without a significant difference compared with the registration assessment without DL (*P* = 0.325). The 6-, 12-, and 24-month LTP rates were 9.9, 20.6, and 24.8%, respectively, in group A, and 4.0, 8.4, and 8.4%, respectively, in group B. There were significant differences between the two groups (*P* = 0.011). Multivariate analysis showed that being >65 years of age (*P* = 0.032, hazard ratio (HR): 2.463, 95% confidence interval (CI), 1.028–6.152) and AM ≤ 5 mm (*P* = 0.010, HR: 3.195, 95% CI, 1.324–7.752) were independent risk factors for LTP after MWA.

**Conclusion:** The novel technology of unsupervised landmark-constrained convolutional neural network-based DIR is feasible and useful in evaluating the ablative effect of MWA for HCC.

## Introduction

Image-guided percutaneous thermal ablation (PTA) is a widely prevalent minimally invasive therapy for early-stage hepatocellular carcinoma (HCC) (1–3). Both microwave ablation (MWA) and radiofrequency ablation (RFA) offer a shorter operative duration, less bleeding, and fewer complications than surgery (4–6). Despite many advantages, the therapeutic effect of PTA is still hampered by local tumor progression (LTP) (7). Accumulating data shows that untreated micrometastases from the primary tumor, the ensuing spread along intrasegmental branches, and vascular invasion can lead to LTP. Previous studies have reported that the LTP rate ranged from 5.1 to 20.7% in patients with a liver malignancy who underwent different ablation modalities (8, 9). Numerous clinical studies have found that a minimum ablation margin (AM) is an independent predictor of LTP after ablation for HCC (10–12). Most micrometastases in previous reports were found to be more than 5 mm away from the boundary of target lesions, and a thermal field range that extends outside the tumor border with a 5–10-mm safe margin should be developed. To improve ablative efficacy, accurate AM assessment would deliver important feedback to the operator during the procedure.

For the assessment of the surgical margin, the margin size refers to the distance from the edge of the neoplasm to the transected tissue (13). Similarly, the AM was measured by the distance among the radiographic borders of the tumor and the ablation zone based on 2D pre- and post-ablative images (14). Traditionally, radiologists typically assess the AM by comparison of the pre- and post-ablation images side by side based on anatomical markers. However, this method fails to measure the AM conveniently and accurately. Instead, pre- and post-ablative images can be registered, and the AM can be measured immediately. However, these registration techniques still have two major issues: first, due to the breathing motion of the liver and heating-stimulated tissue deformation (15, 16), the registration error between the pre- and post-ablation images is augmented; second, there is no specific cutoff value for the optimal safety boundary value. Therefore, precise assessment in the AM and image registration play a vital role in improving the predictive accuracy of LTP after PTA.

Deep learning (DL) is a subspecialty of machine learning that has achieved impressive performance in diagnosis, prediction, and decision-making. In recent years, DL has been applied to image registration and DL-based registration methods can be divided into two categories: one method is to utilize a deep neural network to estimate the similarity between the two images of pre- and post-ablation and drive iterative optimization, and the other method utilizes a deep regression network to predict the transformation parameters. The former methods only use deep learning for the similarity measurement, but they still need the traditional registration method for iterative optimization and cannot perform real-time registration. The latter methods take advantage of DL and address the challenges of non-rigid registration. Balakrishnan et al. (17) proposed an unsupervised learning-based deformable image registration method for MR brain registration. They used a convolutional neural network-based framework, VoxelMorph, to map an input image pair to a deformation field that aligns these images. Zhao et al. (18) presented recursive cascaded networks for deformable image registration. They warped the moving image successively by each cascade recursively in an unsupervised manner and finally aligned to the fixed image.

The goal of this study was to develop and explore AMs using registration between pre- and post-ablation MRI images based on DL, which overcomes the limitations of the current techniques and increases the AM accuracy assessment post-MWA in early-stage HCC.

## Materials and Methods

### Patient Selection

**Figure 1:**
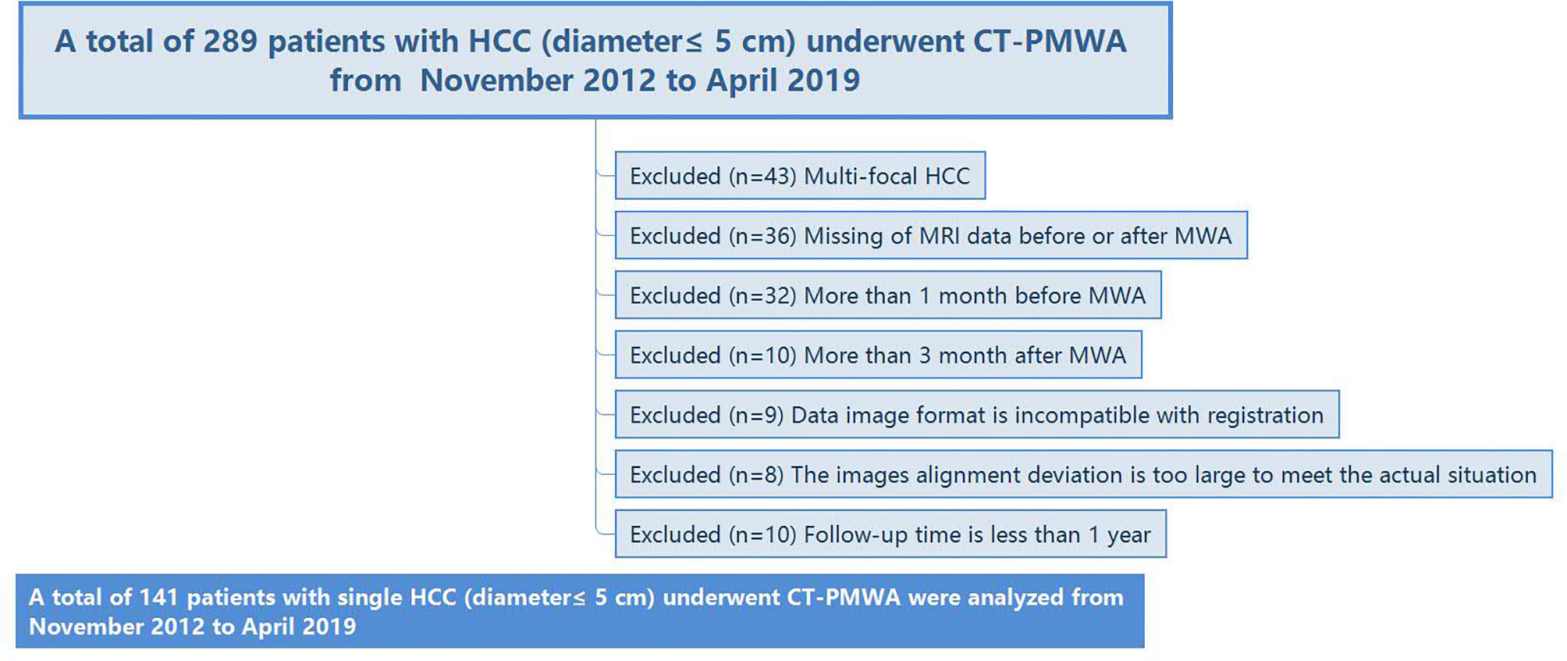
Flow diagram shows the study patient accrual process.

The protocol was reviewed and granted approval through the institutional review board. The necessity to acquire informed consent was waived. For our cohort study, 289 treatment-naïve patients with HCC (tumor diameter ≤ 5 cm) who subsequently were administered computed tomography-guided percutaneous microwave ablation (CT-PMWA) from November 2012 to April 2019 were reviewed. The patients were monitored from time of treatment until death or April 2020. A diagnosis of HCC was established as per recommendations of the European Association for the Study of the Liver (EASL) (19). To avoid any confounding factors that might cause LTP, we designed strict inclusion and exclusion criteria (Figure 1). The inclusion and exclusion criteria are described in Supplementary Material A1. The ablation area covering the tumor focus was examined by comparing the real-time images that were acquired after the procedure with the enhanced scan image that were acquired prior to treatment to confirm complete ablation. The AM is defined as the shortest distance from the edge of the tumor to the edge of the ablation zone. These patients had undergone necessary follow-up examinations.

### Pre- and Post-ablative MRI and Follow-Up

All pre-ablation magnetic resonance image (MRI) scans were performed within 1 month (mean, 12.8 ± 2.2 days; range, 1–29 days) before the MWA procedure. The post-ablation MRI scans were also performed within 3 months (mean, 34.5 ± 10.5 days; range, 25–82 days) following the MWA procedure. Two GE 750w 3.0T MRI scanners were used (GE Healthcare, Waukesha, WI). MRI image parameters used for registration are shown in Supplementary Material A2. Contrast-enhanced MR images were obtained through the use of a T1-weighted 3D gradient-echo sequence prior to and 30, 60, and 90 s post-intravenous administration of 0.1 mmol/kg gadopentetate dimeglumine (Bayer HealthCare Pharmaceuticals, Berlin, Germany). The patients were monitored using a contrast-enhanced imaging (i.e., MRI or CT) at 3-month intervals within 1 year and 6-month intervals beyond 1 year, and the follow-up period was not <1 year.

### MWA Procedures

Ablation was conducted through three interventional radiologists (JH, ZH, and CA, with 25, 10, and 5 years of experience with MWA, respectively). All MWA procedures were carried out under CT guidance, the microwave antenna was localized into the tumor, and the deployment degree scale was established based on the tumor size and shape. Patients were asked to lie in a supine or prone position on the scanning bed based on the location of their lesions. Every MWA procedure was carried out using local and intravenous anesthesia. Post-anesthesia, a 15-gauge, 18-cm MWA antenna (MTC-3C, Nanjing Qinghai Research Institute of Microwave Electric, China) was introduced into the tumor at a pre-set angle. In order to make sure that the position of the ablation electrode was adequate, CT image scanning was carried out once more before the ablation surgery. The settings for the power and ablation times were established as per the standard guidelines that were recommended by the manufacturer. Each MWA session utilized an overlapping technique to make sure the entire tumor was eliminated.

### Definition of Local Tumor Progression and Technique Effectiveness

LTP was characterized based on the imaging results of the abnormal nodular, disseminated, and/or atypical patterns of peripheral enhancement around the ablation site in MWA-treated patients. The efficacy of the technique was described as comprehensive local necrosis at 1 month post-treatment (20).

### Image Registration Procedure

**Figure 2:**
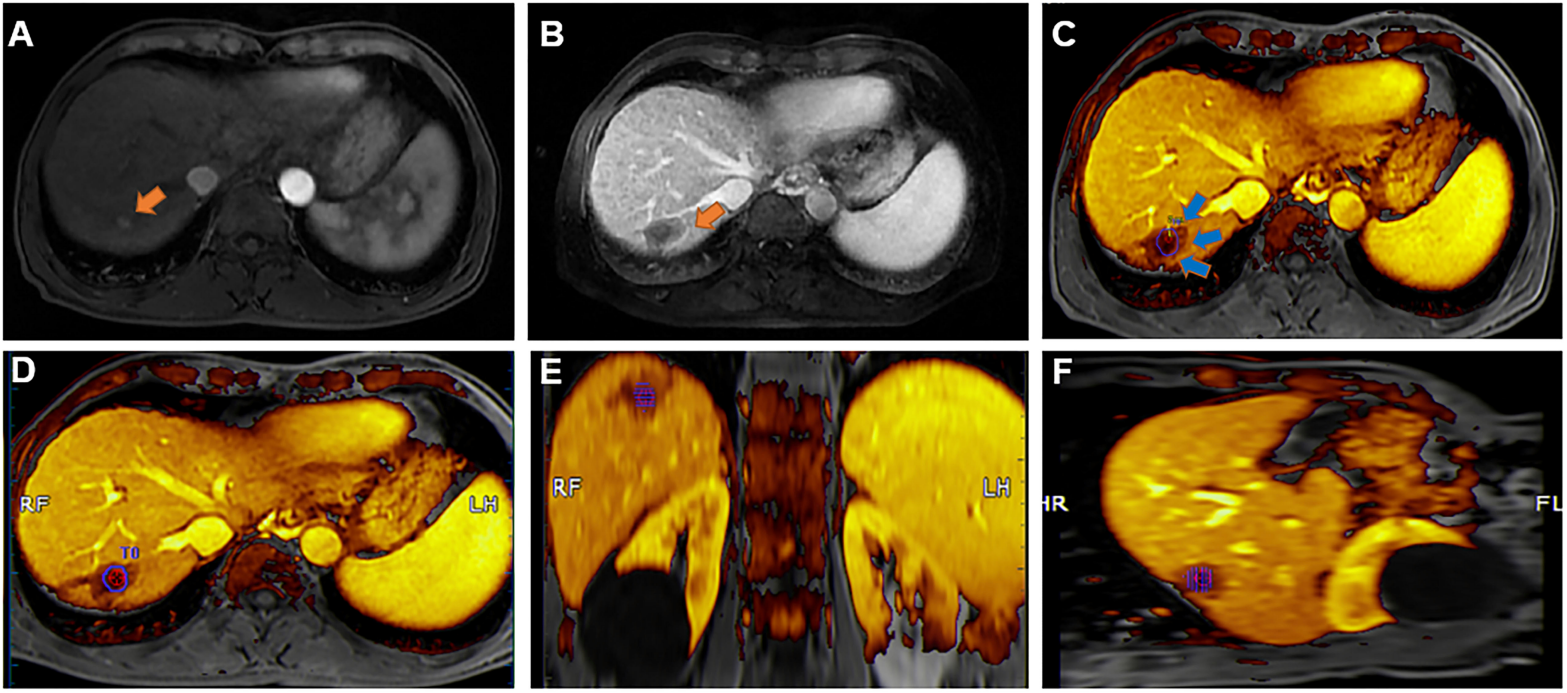
An example of margin assessment based on MR-MR fusion. **(A)** Co-registered pre-ablation MR images show the tumor zone (orange arrow); **(B)** Co-registered post-ablation MR images show the ablation zone (orange arrow). **(C)** Achievement of registration between the tumor and ablation zones (blue arrow). **(D–F)**. Segmented tumor (red) and theoretical 5 mm (blue) margin contours overlaid on the ablation zone on the axial, sagittal, and coronal MR images.

The MRI–MRI image fusion was carried out utilizing a commercial image fusion system (MyLab Twice, Esoate, Genoa, Italy) (11). One set of MRI images prior to MWA that demonstrated hepatic vessels clearly and HCC lesions within the portal vein or delay phase were chosen. Next, the images in DICOM format were imported into the image fusion system. An additional set of MRI images post-MWA with clear hepatic vessels and ablative zones in DICOM format were also imported within the image fusion system. HCC lesions in the MRI scans before MWA were manually outlined, and a 5-mm AM was automatically established. The system labeled the HCC lesion and AM through the use of various colors (Figure 2).

### Deep Learning-Based Deformable Image Registration

**Figure 3:**
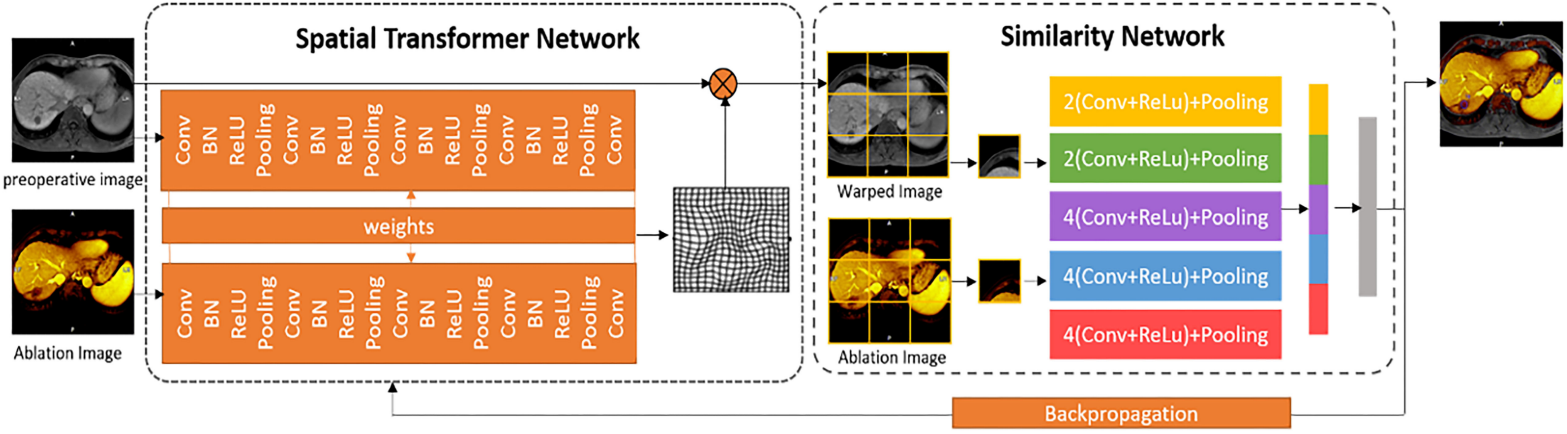
The non-rigid DIR STS-net. Two neural network (left: spatial transformer network and right: similarity network). The orange frame is a non-rigid transformation parameter generation framework in which there are three parts: The localization network specified by a Siamese network, grid generator, and sampler. The blue frame is a similarity calculation framework where the deep multiscale features from the transformed pre-operative image and ablation image are extracted by a convolutional neural network, and their similarity is backpropagated to the orange frame for more accurate registration.

To reduce the registration errors due to breathing motion and heating-induced tissue deformation, we present a deep learning-based deformable image registration (DIR) algorithm based on an unsupervised end-to-end deep spatial transformed similarity network (STS-net) for the ablation images. The architecture of our proposed registration method is given in Figure 3. The registration network, STS-net, contains a spatial transformer network (ST-net) and a similarity network (S-net). The ST-net performs explicit spatial transformations of moving images according to fixed images, and the S-net calculates the similarity between pairs of transformed moving images and fixed images. By backpropagating the similarity from the S-Net, the ST-net can achieve the optimized spatial transformation for unsupervised registration.

The ST-net forms a spatial transformer. First, a Siamese network, which includes two identical convolutional neural networks that share the same set of weights with a final regression layer, is designed to create the non-rigid transformation parameters that minimize the difference between the pair of images. Next, the predicted spatial transformation parameters are utilized to generate a sampling grid to obtain the rigid and non-rigid transformations. Finally, the sampler achieves the warped image sampled from the original moving image at the grid points. For a better transformation, we use the differentiable image sampler, which takes the set of sampling points, and an input image U to produce a transformed moving image V. The transformed image V can be determined using

(1) $$\begin{array}{l} V_{i}=\overset{H}{\underset{n}{\sum }}\overset{W}{\underset{m}{\sum }}U_{nm}\max\left(0,1-|x_{i}-m|\right)\max\left(0,1-|y_{i}-n|\right), \end{array}$$

where *H* and *W* are the height and width of the image, respectively; the (*x*_*i*_, *y*_*i*_) coordinates define the spatial location of pixel *i* in the input image, and max(*a, b*) is a function returning the larger value between value *a* and value *b*. The bilinear sampling kernel is used to obtain the value at a particular pixel in image *V*.

The S-net measures the similarity metric between the paired images. For a more accurate measurement, the whole images are divided into regions, and the similarities between the paired regions are calculated and combined. First, the deep feature is extracted by a convolutional neural network. The regions are passed through a stack of convolutional layers to capture the notion of left, right, up, down, and center, are linearly transformed, and are passed through five max-pooling layers to maintain the local translation invariance property, for a set of feature maps. Then, the multiscale features are fused to form a fully connected layer. Finally, the similarity of deep features from the paired images is calculated in the sum of the similarity between the paired deep features of regions with the normalized cross-correlation. The similarity as the loss function is backpropagated to the ST-net for more accurate registration of ablation images. The loss function can be defined by

(2) $$\begin{array}{l} LOSS=-\overset{N}{\underset{i=1}{\sum }}\frac{D\left(F_{wi},F_{ai}\right)}{\sqrt{D\left(F_{wi},F_{wi}\right)\times D\left(F_{ai},F_{ai}\right)}}, \end{array}$$

where *F*_*wi*_ and *F*_*ai*_ are the features of the *i*th block of the warped image and ablation image, respectively, which are extracted from the fully connected layer, *D* (*a, b*) is a function returning the dot product of vector *a* and vector *b*, and *N* is the number of blocks in the warped image.

The proposed network can learn changes in the position and deformation of the liver according to the pair of the pre- and post-ablation images and apply the transformation on the pre-operative image to obtain the warped image with the deformation. By backpropagating the similarity and correcting the deformation iteratively until the minimum dissimilarity is reached, the pre-operative image is finally aligned to the ablation image.

### Statistical Analysis

Continuous variables were evaluated through the Mann–Whitney *U*-tests, while categorical variables were assessed through the Pearson χ2 or Fisher's exact tests. LTP was then determined utilizing the Kaplan–Meier method using a log-rank test. Univariate and multivariate analyses of independent risk factors for LTP were evaluated using a forward stepwise Cox regression model. The variation in prediction power among the metrics was determined by comparing the area under the receiver operating characteristic curve utilizing DeLong's method. SPSS 22.0 (SPSS, Chicago, IL) and RMS package for the R environment 3.5.1 (http://www.r-project.org/) were utilized for all statistical analyses. A two-sided *P* < 0.05 was the threshold for statistical significance.

## Results

### Baseline Characteristics

After layer-by-layer screening according to the above exclusion criteria, 141 patients (17 females and 124 males; mean age, 55.2 ± 10.8 years) using single HCC (mean diameter, 2.3 ± 0.9 cm) were enrolled. All HCC lesions underwent deep learning-based deformable image registration (DIR), and the success rate of registration was 100% (141/141). The median image registration time cost was 183.5 s, and the mean registration error was 1.6 ± 0.8 mm, which is significantly lower than that of the registration method without DL (2.8 ± 1.1 mm, *P* = 0.003). The patient and tumor characteristics are demonstrated in Table 1. The mean maximum tumor and ablation zone sizes were 2.3 ± 0.9 cm and 3.8 ± 1.2 cm, respectively. The median maximum tumor and ablation zone volumes were 47.8 and 102.6 ml, respectively. The mean minimum ablation margin was 3.4 ± 0.7 mm (range, 0–16 mm). In total, 80 patients successfully achieved a 5-mm safe margin, and 61 patients failed to achieve 5-mm safe margins.

**Table 1:** Baseline characteristics of patients undergoing CT-PMWA.

**Characteristics**	**No. of patients (*n* = 141)**
**Age (y)**^**a**^	55.2 ± 10.8 (26–82)
**Gender**	
Female	17 (12.1)
Male	124 (87.9)
**Comorbid disease**	
Absence	41 (29.1)
Presence	100 (70.9)
**Maximum tumor diameter (cm)**^**a**^	2.3 ± 0.9
**Maximum ablation zone diameter (cm)**^**a**^	3.8 ± 1.2
Tumor volume (ml)^*^	47.8 (16.3–352.8)
Ablation zone volume (ml)^*^	102.6 (76.5–892.6)
**Child–Pugh class**	
A	140 (99.3)
B	1(0.7)
**Location of tumor**	
Left S1/S2/S3/S4	2/2/3/6
Right S5/S6/S7/S8	35/34/24/35
**Abutting major vessels**	
Presence	25 (17.7)
Absence	116 (82.3)
**Biochemical tests**	
AFP (ng/ml)^*^	32.7 (6.3–22352.8)
ALP (U/L)^*^	86.1 (49.0–241.6)
AST (U/L)^a^	36.1 ± 10.8
ALT(U/L)^a^	37.7 ± 11.6
TBIL (μmol/l)^a^	15.6 ± 3.9
**Ablation margin**	
≤ 5 mm	61 (43.3)
>5 mm	80 (56.7)
**Ablation duration (min)**^**a**^	8.7 ± 1.6
**Ablation power (W)** ^**a**^	58.2 ± 1.2

*Unless otherwise indicated, numbers in parentheses are the range*.

a *Values are mean value ± standard deviation (range)*.

* *Values are median (range); AFP, alpha-fetoprotein; AST, aspartate aminotransferase; ALT, alanine transaminase; ALB, serum albumin; TBIL, total bilirubin; MWA, microwave ablation*.

### Comparison Between DIR and Conventional Registration Assessment

Using the conventional registration technique without DL, after registration based on the intrahepatic structure landmark, in three of these cases, significant deviations were visible due to heating-induced tissue deformation. However, 11 cases were misaligned due to breathing motion. If these incorrect registrations were followed, seven patients were considered not to reach the ablation margin. As there is only a limited amount of cases in categories “AM > 10 mm” and “AM = 0 mm,” we divided these patients into two groups for statistical analysis and comparison between DIR and conventional registration without DL. Table 2 shows the comparative results. Compared with registration without DL, DIR was classified into a proportion of ablations as AM ≤ 5 mm (61 vs. 70), and others as margin >5 mm (80 vs. 71). The statistical analysis results demonstrated that the minimum AM calculated utilizing the DIR technique had increased discrimination power for 2-year LTP without a significant difference compared with the registration assessment without DL (AUC, 0.728 vs. 0.705, respectively; *P* = 0.325).

**Table 2:** Comparison between DIR and conventional registration assessment.

	**Conventional registration**				
**DIR**	**AM** **≤** **5 mm**		**AM** **>** **5 mm**		**Total**
	**LTP**	**Non-LTP**	**LTP**	**Non-LTP**	
AM ≤ 5 mm	8	34	8	11	61
AM > 5 mm	3	26	0	51	80
Total	11	60	8	62	141

*AM, ablative margin; DIR, deep learning-based deformable image registration; LTP, local tumor progression*.

### Ablative Margin and Tumor Size

**Figure 4:**
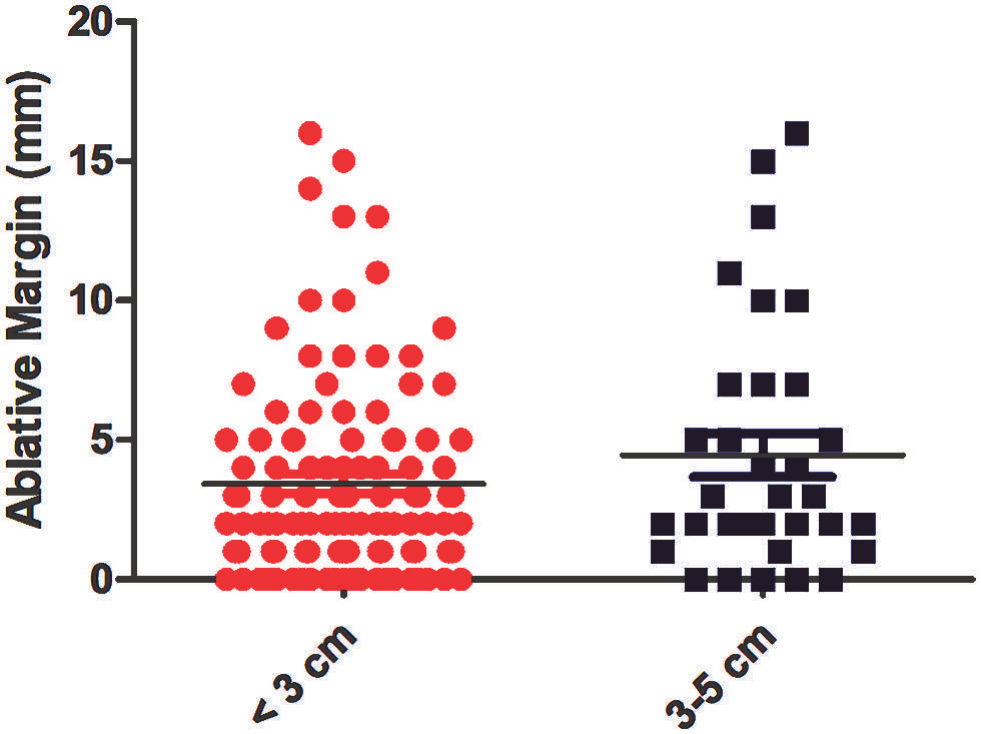
The correlation between the minimal (ablative margin) AM and tumor size. The histogram shows that there is no statistical difference in the AM between the <3-cm group and 3–5-cm group.

According to tumor size, we divided these patients into two groups: the <3-cm and 3–5-cm groups. The mean AM size was similar to that in the 3–5-cm group, demonstrating no significant differences (*P* = 0.403). The correlation between the minimal AM (average of the measured margins by the observers) and tumor size is demonstrated in Figure 4.

### Midterm Local Tumor Progression After CT-PMWA

**Figure 5:**
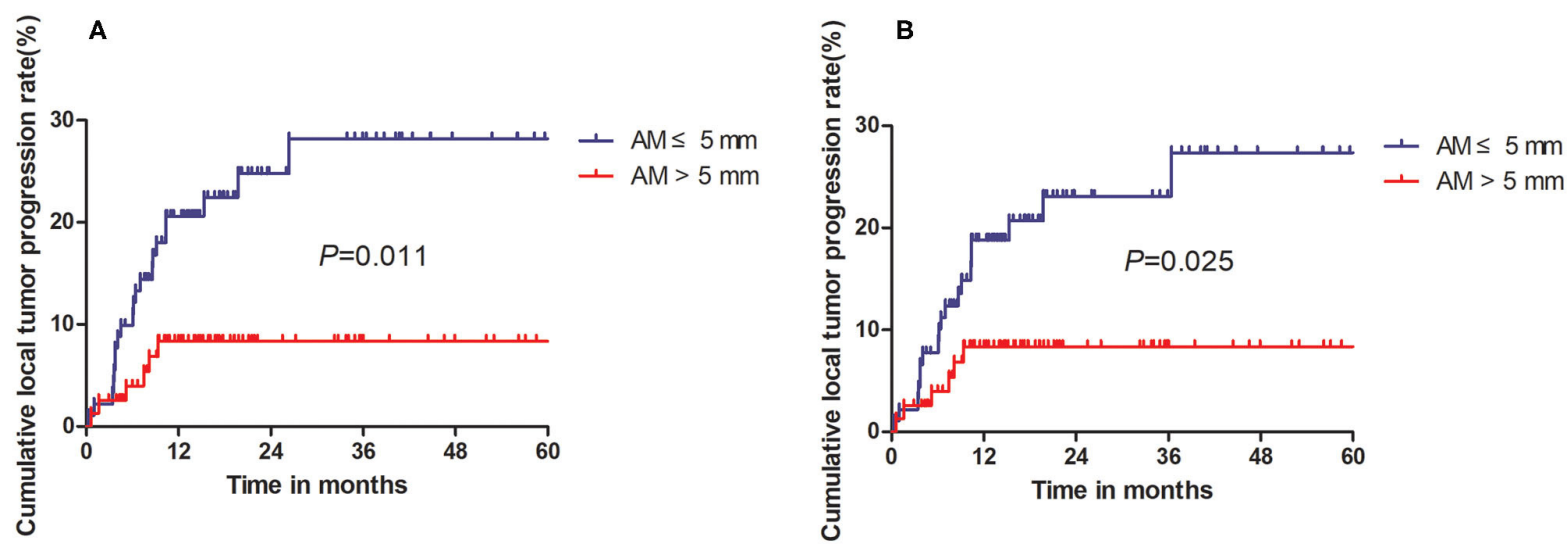
Kaplan–Meier curves comparing local tumor recurrence between the AM ≤ 5-mm and AM > 5-mm groups. **(A)** Comparison of LTP based on deep learning-based deformable image registration (DIR) technique. **(B)** Comparison of LTP based on registration without deep learning.

The median follow-up period was 28.9 months (range, 12.3–89.2 months). In total, 13.5% of patients (19/141) had experienced confirmed LTP. Based on follow-up imaging, the efficacy rate of this technique was 98.6%. These patients were separated into two groups including (1) patients with an ablation area that fully covers the tumor but fails to attain the 5-mm safe margin (group A) and (2) patients with an ablation area that completely covers the tumor and effectively achieves the 5-mm safe margin (group B). With the DIR technique, of the 61 HCC patients in group A, 16 experienced LTP, whereas three patients experienced LTP in group B. In the conventional registration technique, of the 70 HCC patients in group A, 11 were found to have LTP, whereas eight were found to have LTP in group B. According to DIR, the cumulative 6-, 12-, and 24-month LTP rates of group A were 9.9, 20.6, and 24.8%, respectively, for group A and 4.0, 8.4, and 8.4%, respectively (Figure 5A), showing a significant difference (*P* = 0.011) between the groups. According to conventional registration without DL, the cumulative 6-, 12-, and 24-month LTP rates were 7.7, 18.8, and 23.1%, respectively, for group A and 4.0, 8.4, and 8.4%, respectively, for group B (Figure 5B), showing a significant difference (*P* = 0.025) among the two groups.

### Univariate and Multivariate Analyses for LTP

Eight potential risk factors (sex, age, comorbidities, cirrhosis, AFP, tumor size, location abutting major vessels, and AM) for LTP were examined through univariate and multivariate analyses (Table 3). Univariate analysis demonstrated statistical significance between the LTP rates dependent on age [hazard ratio (HR) = 2.891; 95% confidence interval (CI): 1.298, 6.439; *P* = 0.009] and AM (HR = 2.426; 95% CI: 1.081, 5.444; *P* = 0.027). The multivariate analysis showed that older age (HR = 2.463; 95% CI: 1.028, 6.152; *P* = 0.032) and an AM ≤ 5 mm (HR = 3.195; 95% CI: 1.324, 7.752; *P* = 0.010) were significant LTP risk factors.

**Table 3:** Factors associated with LTP according to univariate and multivariate analysis.

**Factors**	**No. of patients**	**Univariate analysis**		**multivariate analysis**	
		**HR (95% CI)**	***P*-value**	**HR (95% CI)**	*P-*value^*^
**Age (years)**		2.891 (1.298, 6.439)	0.009	2.463 (1.028, 6.152)	0.032
<65	113				
≥65	29				
**Gender**		2.839 (0.462, 4.524)	0.513	…	…
Male	124				
Female	17				
**Comorbidities**		2.129 (0.651, 6.961)	0.211	…	…
Absence	41				
Presence	100				
**Cirrhosis**		2.129 (0.651, 6.961)	0.211	…	…
Absence	51				
Presence	90				
**Tumor size(cm)**		1.864 (0.824, 4.219)	0.135	…	…
<3	108				
3–5	33				
**Abutting major vessels**		1.018 (0.501, 2.068)	0.960	…	…
Absence	116				
Presence	25				
**AFP (ng/mL)**		1.585 (0.667, 3.830)	0.292	…	…
≤ 200	112				
>200	29				
**Ablative margin (mm)**		2.426 (1.081, 5.444)	0.011	3.195 (1.324, 7.752)	0.010
≤ 5	61				
>5	80				

*Data in parentheses are 95% confidence intervals*.

* *P-values were determined with Cox proportional hazards regression models. P < 0.05 indicated a significant difference*.

*LTP, local tumor progression; HR, hazard ratio; CI, confidence intervals; AFP, α-fetoprotein*.

## Discussion

Registering the pre- and post-ablation images has been a promising alternative to conventional side-by-side assessment for AMs, which has many advantages as follows (21–23): (1) faster and more accurate measurement of safety boundaries and (2) clearer observation of the spatial relationship between the tumor and ablation zone. Therefore, an increasing number of registration methods have emerged for evaluating AMs. Soichiro Tani et al. (24) reported that a non-rigid intensity-based registration was used to develop a 3D distance map encompassing the tumor and computed the ablation volume to identify the area with insufficient margins. Elena A. Kaye et al. (25) suggested that the generation of new 3D assessment metrics can easily measure the volume of tissue at-risk post-ablation and predicted LTP. However, a crucial issue remains unresolved. The pre- and post-image misalignment of the liver due to the breathing motion and heating-stimulated tissue deformation may result in incorrect AM measurements (26, 27). To effectively reduce this measurement error, we use DL methods for optimal registration in this study.

In this study, we introduced the MRI registration method, which is semi-automated and interactive, utilizing a commercial image-processing software that is utilized in clinic in radiology and interventional oncology. An additional 5-mm boundary beyond the tumor can be automatically calculated and depicted. Although this method can effectively shorten the AM assessment time and decrease the evaluation bias of different radiologists, it is still unable to achieve the most accurate registration between the tumor and ablation zone. The difficulty of traditional registration methods is the design of similarity measures and the selection and matching of features. The unsupervised DL-based registration method proposed in this paper can use the derivable spatial transformer to optimize the image similarity between pre- and post-ablative MRI images. Our method does not avoid the extraction of handcrafted features, the matching design, and the similarity measure, and it also uses extensive clinical data that has not been annotated by medical experts. In addition, our DIR method uses the Siamese spatial transformer network to obtain the non-rigid transformation parameters more accurately than other methods and uses backpropagation to continuously optimize the similarity of the paired pre- and post-ablative images to minimize the distance between them (28–30). The proposed registration method can make full use of the advantages of deep neural networks to achieve better registration performance than previous methods.

The obtained AM in patients with solitary HCC from CT-PMWA were analyzed. Of the 144 patients, 7.8% of patients (11/141) patients had suboptimal co-registration results from differences in liver position and/or shape. Interestingly, all the suboptimal co-registrations were improved by DL and eventually reached sufficient integration. Because several patients had inaccurate AMs when using the conventional registration technique, the predictive power of the AM for LTP may weaken. However, the DIR technique has more powerful prediction ability than conventional registration techniques based on better AUC values for the prediction of LTP.

In this study, there were three major findings. First, using DL-based registration can improve the predictive power. The higher AUC value compared with the conventional registration technique and the cumulative LTP rate of patients in the ≤ 5-mm AM group being significantly increased compared to the >5-mm AM group can explain the advantage of DL-based registration; second, the minimal AM size was not affected as the tumor diameter increased when patients underwent CT-PMWA, and the reason may be that MWA can generate a larger ablative zone easily; third, in addition to the AM, older age (>65 years old) was also a risk factor for LTP and deserved our attention before ablation treatment.

Our study has several limitations. First, assessment of the technique utilizing the diagnostic pre- and post-ablation MRI images with a 5-mm slice thickness limits the accuracy of the slice direction assessment. Optimally, further studies should try to acquire thinner slices. Secondly, the DIR of potential value for intra-ablation use would require a prospective study in an HCC patient cohort with similar characteristics. In fact, post-ablation imaging will possibly be obtained using the ablation applicator that remains in the tissue, which introduces a degree of beam hardening artifact that impacts segmentation performance. Third, our study design is a limitation, as the person evaluating the novel technique was not blinded to the LTP-associated outcomes and may be subjected to bias. Future studies will be focused on the assessment and adjustment of this technique for intraprocedural utilization. Final, no blinded valued method may cause biases and the larger sample and further perspective studies can be needed.

In conclusion, non-rigid DIR permits us to quantitatively assess the adequacy of the AM post-CT-PMWA. This method can help predict LTP at an earlier time point, including immediately after the ablation procedure and lead to an improvement in patient care.

## Data Availability Statement

The datasets used and/or analyzed during the current study are available from the corresponding author on reasonable request.

## Ethics Statement

The studies involving human participants were reviewed and approved by the institutional review board of Sun Yat-sen University Cancer Center. The patients provided their written informed consent to participate in this study. Written informed consent was obtained from the individual(s) for the publication of any potentially identifiable images or data included in this article.

## Author Contributions

CA participated in the data analysis and drafted the manuscript. JH and YG conceived of the study and carried out the editorial support for this manuscript. JH, ZH, and TZ participated in the ablation procedure. LM provided deep learning-based deformable image registration method. YJ provided assistance in data analysis. All authors read and approved the final manuscript.

## Conflict of Interest

The authors declare that the research was conducted in the absence of any commercial or financial relationships that could be construed as a potential conflict of interest.
