# Supplementary material for: Assessment of Ablative Margin After Microwave Ablation for Hepatocellular Carcinoma Using Deep Learning-Based Deformable Image Registration
Source: Front Oncol. 2020 Sep 24;10:573316. doi: 10.3389/fonc.2020.573316 (PMC7546854; doi:10.3389/fonc.2020.573316)
Supplement: Supplementary file 1 [file Data_Sheet_1.docx]

**The inclusion and exclusion criteria**

The inclusion criteria were as following: (1) patients aged older than 65 years; (2) patients with Eastern Cooperative Oncology Group (ECOG) performance status 0 or 1; (3) patients with Child-Turcotte-Pugh (CTP) grades A or B; (4) patients with a single tumor sized < 5 cm; (5) patients without major vascular infiltration or extrahepatic metastasis.

The exclusion criteria were utilized to determine the final cohort for this study, including: (1) follow-up time is less than 1 year; (2) multi-focal HCC; (3) missing of MRI data before or after MWA; (4) data image format is incompatible with registration; (5) the images alignment deviation is too large to meet the actual situation; (6) preoperative diagnosis time is more than 1 month; (7) postoperative assessment time is more than 3 months.
