# Supplementary material for: Assessment of Ablative Margin After Microwave Ablation for Hepatocellular Carcinoma Using Deep Learning-Based Deformable Image Registration
Source: Front Oncol. 2020 Sep 24;10:573316. doi: 10.3389/fonc.2020.573316 (PMC7546854; doi:10.3389/fonc.2020.573316)
Supplement: Supplementary file 2 [file Data_Sheet_2.docx]

**MRI parameter**

Imaging included an axial T1-weighted fast field echo (FFE), axial T2-weighted single-shot turbo spinecho (SSTSE), axial in-phase and out-phase chemical shift GRE T1-weighted images, and a gadolinium enhanced dynamic study. The parameters of FFE

were as follow: repetition time/echo time, 129/238 and 476 ms; number of sections, 60; field of view, 380 mm; matrix, 158 × 256; flip angle, 70°; gap, 15%; section thickness, 3 mm; two signals acquired. The parameters of SSTSE were as follow: repetition time/echo time, 2100/84 ms; number of sections, 60; field of view, 350 mm; matrix, 207 × 384; flip angle, 150°; gap, 10%; section thickness, 3 mm; one signal acquired. For the gadolinium enhanced dynamic study, a multiphase dynamic study including arterial, portal, and delayed phases was performed before unenhanced MR imaging. The parameters of enhanced study were as follow: repetition time/echo time, 3.89/1.51

ms; number of sections, 52; field of view, 420 mm; matrix, 144 × 384; flip angle, 25°; gap, 20%; section thickness, 3 mm; one signal acquired.
